# Universal dependence of the spin wave band structure on the geometrical characteristics of two-dimensional magnonic crystals

**DOI:** 10.1038/srep10367

**Published:** 2015-05-27

**Authors:** S. Tacchi, P. Gruszecki, M. Madami, G. Carlotti, J. W. Kłos, M. Krawczyk, A. Adeyeye, G. Gubbiotti

**Affiliations:** 1 Istituto Officina dei Materiali del CNR (CNR-IOM), Unità di Perugia, c/o Dipartimento di Fisica e Geologia, Perugia, Italy; 2 Faculty of Physics, Adam Mickiewicz University in Poznan, Umultowska 85, Poznan 61-614, Poland; 3 Dipartimento di Fisica e Geologia, Università di Perugia, Italy; 4 Information Storage Materials Laboratory, Department of Electrical and Computer Engineering, National University of Singapore, 117576 Singapore

## Abstract

In the emerging field of magnon-spintronics, spin waves are exploited to encode, carry and process information in materials with periodic modulation of their magnetic properties, named magnonic crystals. These enable the redesign of the spin wave dispersion, thanks to its dependence on the geometric and magnetic parameters, resulting in the appearance of allowed and forbidden band gaps for specific propagation directions. In this work, we analyze the spin waves band structure of two-dimensional magnonic crystals consisting of permalloy square antidot lattices with different geometrical parameters. We show that the frequency of the most intense spin-wave modes, measured by Brillouin light scattering, exhibits a universal dependence on the aspect ratio (thickness over width) of the effective nanowire enclosed between adjacent rows of holes. A similar dependence also applies to both the frequency position and the width of the main band gap of the fundamental (dispersive) mode at the edge of the first Brillouin zone. These experimental findings are successfully explained by calculations based on the plane-wave method. Therefore, a unified vision of the spin-waves characteristics in two-dimensional antidot lattices is provided, paving the way to the design of tailored nanoscale devices, such as tunable magnonic filters and phase-shifters, with predicted functionalities.

The possibility of using spin waves (SWs) in artificially modulated magnetic media, named magnonic crystals (MCs), as a new means of signal processing, communication and computation over a very large bandwidth, stimulated the emerging research field of “magnonics” or “magnon-spintronics”^1^,^2^. Here, the information can be encoded in both the amplitude^3^ or the phase^4^ of SWs, enabling the design of a new class of wave-based ICT devices. Compared to conventional microwave devices, those based on SWs can be orders of magnitude smaller, as SWs operating at GHz or THz frequencies have micrometric or nanometric wavelength, i.e. much shorter than corresponding electromagnetic waves. The SW spectrum in MCs is characterized by frequency bands and forbidden frequency band-gaps whose position can be magnetically tuned^5^,^6^,^7^,^8^,^9^,^10^,^11^,^12^,^13^. Therefore, MCs represent the magnetic counterpart of photonic^14^, phononic^15^ and plasmonic^16^ crystals, with peculiar features connected to the nonreciprocal properties of spin waves^17^,^18^,^19^,^20^, i.e. characterized by asymmetric dispersion relation [*ω*(***k***) ≠ *ω*(−***k***), where *ω* is the angular frequency and ***k*** is a wave vector], which makes them useful for microwave isolators and circulators. The interest toward MCs also results from their potential use as high-sensitivity magnetic sensors^21^, tunable filters or phase shifters^22^,^23^ and transistors^24^. Most of the previously proposed devices consist of one- and two-dimensional (1-D and 2-D) yttrium iron garnet (YIG) films with an artificial micron-size periodicity and SWs generated by inductive methods^25^. YIG has the advantage of being a very low-damping material, but its integration into standard semiconductor technology is challenging due to the difficulties of growing and patterning high quality thin films on substrates different from gadolinium gallium garnet (GGG) single crystals. Polycrystalline magnetic alloys such as NiFe (Permalloy) or CoFeB, would be better suited to meet to the industrial demand of miniaturization and integration, but their drawback is the relatively high SW damping which prevents propagation above a few microns. However, this can be compensated by downscaling the MC to the nanometric size, also thanks to the new possibility of launching SWs with sub-micrometric wavelength by spin-transfer torque^26^ or spin Hall effect^27^. Therefore, 2-D MCs obtained from metallic alloys are being considered for the next generation of the above mentioned applications^23^,^28^,^29^,^30^,^31^.

In this work we exploit the Brillouin light scattering (BLS) technique to study the SWs spectrum of 2-D MCs consisting of square antidot lattices (ADLs), i.e. arrays of holes drilled into a magnetic Permalloy film. From previous studies, it is known that such ADLs can be considered as a network of multiple connected magnonic waveguides having an effective width equal to the distance between neighboring hole edges, where both confined and propagating spin waves coexist^32^,^33^,^34^,^35^,^36^. However, an overall comprehension of the relationship between the geometric characteristics of the ADLs and the SWs properties is still lacking. By investigating the SWs dispersion characteristics of several square ADLs with different thickness (*t*) and inter-hole separation (*w*), we found that the frequency of the most relevant modes at Γ point (*k*_*y*_ = 0), i.e. at the center of the Brillouin Zone (BZ), as well as the frequency position and the width of the band-gap at the BZ boundary (Y point, i.e. *k*_*y*_ = π/*a* ) exhibit a universal dependence on the aspect ratio *t/w* of the ADL.

The experimental results are successfully interpreted on the basis of calculations performed by the plane wave method (PWM), taking into account both dipolar and exchange interactions^37^. This general and unified view that governs the spin-wave characteristics in squared ADLs has not been previously identified, and offers useful information for predictive purposes and device design operating in the microwave frequency range.

## Results and discussion

### Samples properties

**Figure 1:**
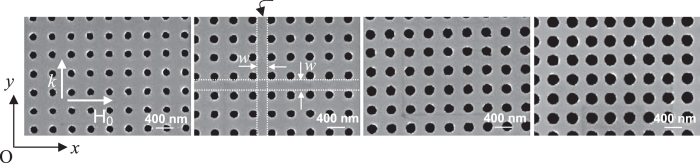
Scanning electron microscopy images of the ADLs samples in Series 3.

Three series of Permalloy (Ni_80_Fe_20_) antidot lattices (ADLs), consisting of circular holes arranged in a square matrix with periodicity *a*, have been studied. Series 1 (S1) and Series 2 (S2) consist of three arrays having fixed hole’s diameter *D* and hole-to-hole distance *w*, but different thickness, ranging between 12 and 30 nm, as illustrated in the Table 1. Series 3 (S3) consists of four arrays having fixed thickness *t* = 30 nm and periodicity *a* = 420 nm, but different diameters (hole-to-hole distance). Scanning electron microscopy images of the ADL samples in S3 are shown in Fig.1. In the same Figure we have also plotted the coordinate system that will be used in the following, with the *x* and *y* axis parallel to the main axis of the ADL.

### Frequency and spatial character of the SW modes

**Figure 2:**
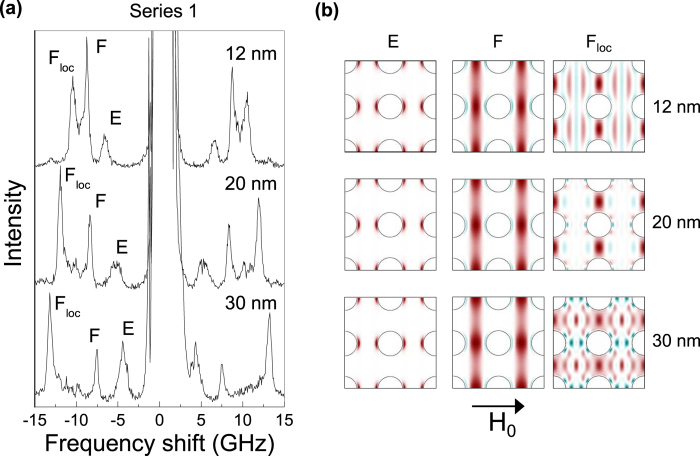
Spin-wave modes at the Γ-point. (a) BLS spectra taken at the Γ-point for the series S1 ADLs with different thicknesses applying a magnetic field μ_0_H_0_ = 0.1 T. **(b)** Calculated SW spatial profiles for the edge (E), the fundamental (F) and the fundamental-localized (F_loc_) modes. The intensity of the color denotes the amplitude of the excitation, while the red and blue colors indicate opposite phase.

Figure 2 shows representative BLS spectra relative to the three S1 specimens, taken at the Γ-point under a magnetic field μ_0_*H*_0_ = 0.1 T applied along the *x-*direction (Fig.1b). Each spectrum is characterized by the presence of three dominant peaks whose frequency and relative cross-section noticeably depend on the ADL thickness. The corresponding modes have been classified on the basis of their frequency and spatial symmetry, comparing the measured spectra to the PWM calculations (Fig. 2b). The low-frequency mode corresponds to the so-called edge (E) mode. This is confined in the deep wells of demagnetizing field appearing close to the holes edges in the direction of H_0_. At higher frequency, the fundamental (F) mode, characterized by a quasi-uniform spin precession amplitude extending in the vertical channels comprised between rows of holes, and the localized fundamental (F_loc_) mode, having the maximum amplitude in the horizontal channels between rows of holes, are found. In the frequency range between the F and F_loc_ mode, several other peaks of low intensity, corresponding to higher order modes, are observed.

**Figure 3:**
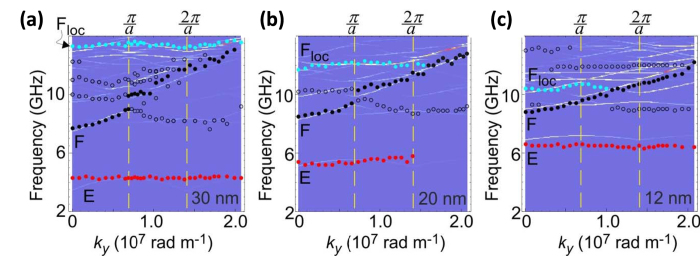
Spin-wave frequency dispersion as a function of the ADL thickness. Comparison between measured (points) and calculated (color plot) spin wave frequency dispersion along the ΓY direction, for samples of thicknesses: **(a)** 30 nm, **(b)** 20 nm, and **(c)** 12 nm from Series 1. The magnetic field μ_0_H_0_ = 0.1 T is applied along the *x*-direction. The full red, black and blue dots mark the E, F and F_loc_ modes, respectively. The intensity of the lines indicates the calculated BLS intensity of the respective modes. The vertical dashed lines show the BZs borders.

In Fig. 3 the comparison between the SW frequency dispersion (points) measured along the ΓY direction of the reciprocal lattice and PWM calculations (color plot) is reported for the three S1 samples. All ADLs are characterized by a very rich band structure which is upshifted in frequency on decreasing *t.* It should be noted that the frequency of both the E and the F modes increases on decreasing the ADL thickness, while that of the F_loc_ mode follows an opposite trend. In addition, only the F mode exhibits a dispersive behavior, with a group velocity (*v*_g_ = *∂ω*/*∂k*), extracted at *k*_*y*_ = 0, of *v*_g_ = 1.15 km/s, 0.67 km/s and 0.29 km/s, for *t* = 30 nm, 20 nm and 12 nm, respectively. This reflects the corresponding dependence of the group velocity of the Damon-Eshbach mode of a continuous films on its own thickness, starting from *v*_g_ = 2.77 km/s (for *t* = 30 nm) to *v*_g_ = 2.30 km/s (for *t* = 20 nm) and finally to *v*_g_ = 0.9 km/s (for *t* = 12 nm). At the Y-point, the Bragg diffraction of the F mode induces the opening of a band gap^38^, whose amplitude diminishes on decreasing *t,* until for *t* = 12 nm the band gap is not experimentally resolved due to the limited frequency resolution of the BLS apparatus. PWM calculations also predict a slight dispersive behavior for the E mode, not visible in the measurements within the experimental accuracy, with *v*_g_ (extracted at *k*_*y*_ = 0) decreasing from 1.15 km/s for *t* = 30 nm to 0.29 km/s for *t* = 12 nm.

### Universal dependence of the modes frequency on the *t/w* ratio

**Figure 4:**
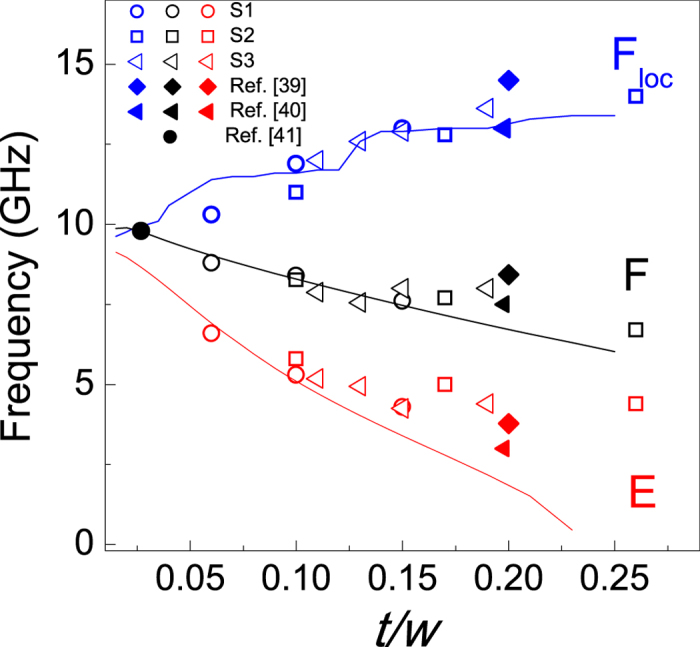
Frequency of the most intense modes, at the Γ-point, as a function the aspect ratio *t/w* of the effective nanowires, comprised between adjacent rows of holes. Measured (open points) and calculated (lines) frequencies of the E (red), F (black) and F_loc_ (blue) modes in dependence on *t/w* ratio for the three Series of investigated samples. Frequencies of the E, F, and F_loc_ modes taken from literature (full points) are also reported.

**Figure 5:**
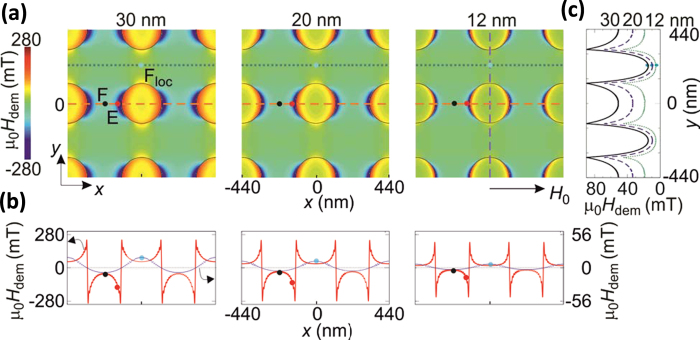
Calculated demagnetizing field as a function of the ADL thickness. (**a**) Demagnetizing field for Series 1 samples, calculated by PWM applying a magnetic field μ_0_H_0_ = 0.1 T along the *x*-direction. The full dots in red, black and blue point at the area of concentrating the SW amplitude of the E, F and F_loc_ mode, respectively. **(b)** Cross-section of the demagnetizing field calculated along the horizontal lines of the panel (**a**). Note that different scales are used for the demagnetizing fields along different cross-sections. **(c)** Cross-section of the demagnetizing field calculated along the vertical line of the panel (**a**).

To elucidate the effect of the ADL geometrical parameters on the band structure, the frequencies of the E, F, and F_loc_ modes, measured at the Γ-point, are plotted in Fig. 4 versus the aspect ratio (*t*/*w*) of the effective nanowires (E-NWs) enclosed between adjacent rows of holes forming the ADL. Interestingly, the frequencies of the different samples present a similar evolution: on increasing *t/w,* a monotonic decrease (increase) of the frequency of the both the E and F mode (F_loc_ mode) has been found. Such a universal scaling is also followed by the frequencies of the E, F, and F_loc_ modes measured in previous works on permalloy ADLs, that are also reported in Fig. 4^39^,^40^,^41^. This indicates that the frequencies do not depend on the geometrical parameters of the ADL separately, but only on the ratio *t/w.* Remarkably, it can be seen that the whole set of these experimental results is well fitted by the curves obtained using PWM calculations, where the antidot diameter *D* was fixed to 240 nm as in S1, while the thickness was varied in the range from 1 to 44 nm. A significant discrepancy between the theoretical curves and the experimental points is only seen for the E mode at large *t/w* values. This is because in the PWM calculations a uniform distribution of static magnetization around the holes is assumed, but this is not fulfilled for large thicknesses where the frequency of the E mode is underestimated. Finally, the presence of steps in the calculated curve of the F_loc_ mode as a function of *t/w* are associated with the anti-crossing of this mode with different SW excitations, densely occupying this frequency range^42^,^43^. The universal scaling of the modes frequency with the aspect ratio can be explained taking into account the evolution of the internal magnetic field in different ADL spatial regions, where the modes are localized (Fig. 5). In particular, according to the calculated spatial profiles reported in Fig.2(b), the E and F modes can be considered as the resonances of transversely magnetized E-NWs enclosed between the ADL columns of holes^44^,^45^, whereas the F_loc_ mode is the lowest resonant mode, with quasi-uniform spatial profile, of a longitudinally magnetized E-NWs enclosed between horizontal rows of holes^46^.

In the limit of *t/w* approaching 0, the frequencies of the F and F_loc_ modes converge towards the same value^47^, which is close to that of the Kittel mode of the continuous film (9.3 GHz). This can be explained observing that, in the central portion of both the longitudinally and transversely magnetized E-NWs, when *t* decreases the demagnetizing field becomes more homogenous and decreases down to zero (Fig. 5(c)). As a consequence the average value of the internal magnetic field comes close to the value of the external bias field. Moreover, on decreasing the ADL thickness the wells of the demagnetizing field at the edges of the holes shrink and become less deep. This results in expelling the localization of the E mode far apart the edges, as it can be observed in the SW profiles shown in Fig. 2(b). As a consequence the distribution amplitude of E mode approaches that of the F one and its frequency is upshifted toward that of the Kittel mode. However, for the E mode, the scenario in the limit *w* → ∞ is different from *t* → 0, because at sufficiently large separation of holes the demagnetizing field does not change significantly with further increase of *w*, thus preserving the frequency of E mode at the same level. Nevertheless, for large values of *w* the cross-section of the E mode is expected to be strongly reduced, due to the corresponding decrease of its extension if compared to the whole area.

In the opposite limit, for large *t/w* values, the wells of the demagnetizing field near the hole edges become deeper. Therefore, the internal magnetic field is reduced, inducing the decrease of the E-mode frequency. Similarly, the demagnetizing field in the central area of the transversely magnetized E-NWs becomes more negative on increasing the ratio *t*/*w,* although to a lesser extent compared to the edges (see, Fig. 5(c)), so the F mode frequency exhibits a less pronounced reduction as a function of *t*/*w*. On the contrary, both an increase of *t* and a reduction of *w* cause a slight increase of the demagnetizing field in the longitudinally magnetized E-NWs, leading to the gradual growing of the frequency of F_loc_ mode as a function of *t/w*. However, we estimated that the increment of the static demagnetizing field in the region where the F_Loc_ mode is localized, when *t/w* passes from 0.06 to 0.15, should cause a frequency increase of the F_loc_ mode of only 0.5 GHz. The larger frequency increase observed in both the experiment and numerical calculations indicates that also the variation of the dynamical demagnetization field with *t/w* gives a substantial contribution to the frequency evolution of the F_loc_ mode. This effect is in agreement with previous findings in longitudinally magnetized stripes with various aspect ratios (where the effects of static demagnetization field are absent)^48^.

### Dependence of the band-gap position and amplitude on *t/w*

**Figure 6:**
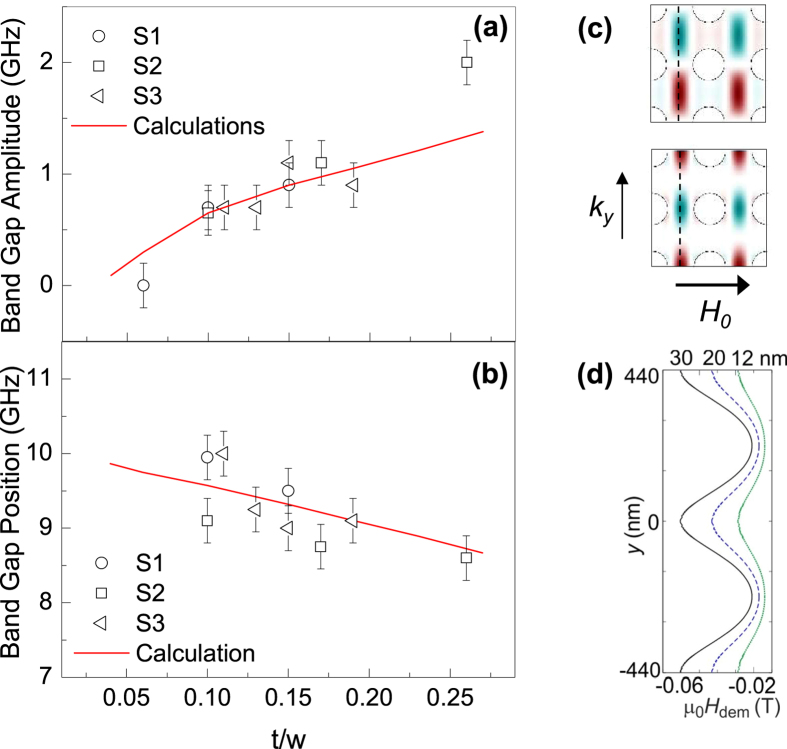
Properties of the band gap at the BZ boundary. (**a**) Band gap amplitude and (**b**) position for the F mode as a function of the *t/w* at the BZ boundary. Points are the BLS data from different Series, and the solid lines are the PWM results. (**c**) Representative spatial profile of the stationary waves, at the border of the first BZ along *Y* direction, calculated for the sample 12 nm thick in S1. (**d**) The demagnetizing field along the central line of the vertical E-NW (black dashed line marked in (**c**)) for 12 (green dotted line), 20 (blue dashed) and 30 nm (solid black) thick samples S1.

The above analysis demonstrated that the more dispersive (and therefore propagative) mode in ADLs is the F mode. Any device based on propagating SWs should therefore make use of the F mode, so that it is worth to analyze the frequency-position and amplitude of the main band-gap, occurring at the boundary of the first BZ (Y-point). Figure 6 shows the measured (points) and calculated (lines) amplitude (a) and position (b) of the band gap for the three investigated Series. It can be seen that, in analogy to the data of Fig. 4, also these quantities follow a universal dependence upon the *t*/*w* ratio. In particular, a monotonic increase (decrease) of the band gap amplitude (position) has been observed on increasing *t/w*. This behavior can be understood taking into account the spatial profile of the two (bottom and top) dispersion branches of the F mode at the Y-point, whose spatial profiles are shown in Fig. 6(c) for the 12 nm thick sample in S1 (the profiles for other samples, not shown here, have a similar amplitude distribution). The two mode-branches are localized into the channels enclosed between the vertical rows of holes and possess an oscillating amplitude along the wave vector direction. Their spatial distribution has periodicity 2*a,* with maxima shifted by *a*/2 relative to each other, in analogy to the well-known case of Bragg reflection of electrons in a periodic potential^49^. The spatial amplitude of the lower-frequency branch is concentrated between adjacent holes, while the amplitude of the higher-frequency branch is concentrated in the space between holes. Looking at the demagnetizing field in the areas occupied by these two branches (see Fig. 6(d)), the difference between the demagnetizing fields in these two areas increases at increasing *t*, resulting in a larger gap width. On the whole, the position of the band gap follows the same dependence on *t/w* ratio as the frequency of F mode in the BZ center (Fig. 3), however the decrease is much slower for the band gap position. This is due to the contribution of the group velocity of F mode, which increases with increasing ADL thickness and affects the band gap position oppositely to the effect of demagnetizing field.

## Conclusion

In conclusion, by measuring spin-wave frequency dispersion in ADLs of different thickness *t* and hole’s separation *w*, we found that the frequency of the most intense modes, measured at the Γ-point, follows a universal law and depend exclusively on the aspect ratio *t/w* of the effective nanowires, comprised between adjacent rows of holes. A similar universal dependence on *t/w*, also governs both the width and the frequency position of the band gap of the fundamental propagating mode. These results provide a unified vision of spin wave characteristics in two dimension magnonic crystals consisting of squared ADLs, in terms of the geometric parameters of the array. Therefore, we are confident that this work will stimulate and facilitate the design of magnonic crystals with tailored functionalities, in view of their application in the next generation of microwave devices.

## Methods

### Fabrication

Large area (4 × 4 mm^2^) antidot lattices (ADLs) with circular holes arranged into a square matrix were fabricated on commercially available Si substrates using deep ultraviolet lithography at 248 nm exposing wavelength, followed by e-beam deposition of Permalloy (Ni_80_Fe_20_) films and ultrasonic assisted lift-off. Three different series of ADLs, whose geometrical parameters are summarized in Table 1, have been prepared and studied. Series 1 (S1) and Series 2 (S2) are characterized by comparable lattice periodicity *a*, but different values of *D* and *w*, i.e. *D* = 240 nm, *w* = 200 nm and *a* = 440 nm (*D* = 355 nm, *w* = 115 nm and *a* = 470 nm) in S1 (S2). For both S1 and S2, three different antidot thicknesses, *t* = 12, 20, and 30 nm, have been analyzed. Series 3 (S3) consists of four arrays having fixed thickness *t* = 30 nm and lattice constant *a* = 420 nm, but different diameters (hole-to-hole distance) comprised in the range between 140 and 260 nm (280 and 160 nm). The corresponding edge of the first Brillouin zone (Y point) at π/*a* are 0.71 × 10^7^ rad/m, 0.67 × 10^7^ rad/m, 0.75 × 10^7^ rad/m for Series 1, 2, and 3 respectively. Continuous (unpatterned) permalloy films with thicknesses of 12, 20 and 30 nm were also prepared and used as reference samples.

### Measurements

Brillouin light scattering spectra from thermally activated spin waves were measured in the back-scattering configuration. The light source is a Coherent VERDI single-frequency, diode-pumped solid-state laser, operating in the spectral line of λ_Laser_ = 532 nm, with a line width of approximately 10 MHz. About 200 mW of laser power are focused upon the sample surface and the light scattered from the sample is analyzed in frequency by using a high-contrast Sandercock-type (3 + 3)-tandem Fabry-Pèrot interferometer^50^. The incident light is *p*-polarized while an analyzer put at extinction, rotated by 90°, with respect to polarization of the incident light, is used at the entrance of the interferometer to reduce the noise level and to suppress signals from acoustic phonons. A magnetic field μ_0_*H*_0_ = 0.1 T (sufficient to fully saturate the sample) was applied parallel to the *x*-direction of the ADL, while the in-plane wave vector *k*_*y*_ of the SWs probed in the experiment was swept along the in-plane perpendicular direction (Voigt or Damon-Eshbach configuration). Due to the photon-magnon conservation law of momentum in the scattering process, the magnitude of the wave vector is linked to the incidence angle of light (*θ*) and to the light wavelength by the simple relation: *k*_*y*_ = (4π/λ_Laser_) × sin(*θ*). In our experiment *k*_*y*_ is varied in the range between 0 and 2.0 × 10^7^ rad/m. This corresponds to map the dispersion curves up to the third Brillouin Zone along the ΓΥ direction of the reciprocal lattice.

### Theoretical Model

To calculate the spectrum of SW in ADLs we solve the linearized Landau-Lifshitz equation in the frequency domain assuming the saturation magnetization in the plane of ADL and a homogeneous distribution of the magnetization dynamics across the slab’s thickness. The assumption of homogeneity across the ADL is justified for considered samples in the investigated frequency range due to large ADL period with respect to its thickness^51^. To solve the eigenproblem and to find the spectrum of frequencies and the spatial profiles of dynamical components of magnetization, we use plane wave method (PWM)^52^,^53^. PWM calculations were supplemented by micromagnetic simulations (MS) performed with the Mumax package^54^ at the center of the Brillouin Zone (*k*_*y*_ = 0). Both, PWM and MS calculations take into account the exchange and dipolar interactions and assume uniform field distribution across the thickness of ADL. The application of such an extended model is necessary for considered regime of dipole-exchange spin waves. PWM is used to calculate full magnonic band structure but it requires to make few further approximations. In particular, the spin wave dynamics is pinned at the edges of the antidots^55^,^56^. To suppress the pinning effect in PWM calculations we added an artificial ring, *d*_r_ = 11.5 nm wide, concentric to ADL holes, with an anisotropy field opposite to the external magnetic field. By means of auxiliary micromagnetic simulations we showed that the presence of surface anisotropy at the edges of the ADL hole is the physical mechanism responsible for the pinning effect. The effective magnetic anisotropy field, having a source in the surface anisotropy, can be calculated as *H*_ani_ = 2 *K*_S_/(μ_0_ *M*_S_ *d*_r_), where *K*_S_ is a surface anisotropy constant (in standard approach this is divided by a film thickness if anisotropy on the film surface is considered). In MS we used a mesh with in-plane size of 1 nm × 1 nm so that we can reduce anisotropy to the single mesh cell located at the hole edge. Thus *K*_S_ was fixed to the value assumed in PWM and concentrate all ‘pinning’ in the cell of 1 nm × 1 nm, we need to decrease *d*_r_ 30 times and increase 30 times *H*_ani_ (it gives anisotropy field of 3 T), which shell be attributed to this mesh cell at the antidot edge. With these MS we have obtained the same frequencies as that from the PWM method without the ring (we have checked also that by taking the mesh of 2 nm × 2 nm in size the agreement with PWM is obtained by assumed 1.5 T anisotropy field). The physical parameters used in the calculations were obtained by a fit of the BLS experimental dispersion measured from the homogeneous permalloy film, 30 nm thick: saturation magnetization M_s_ = 0.8 × 10^6^ A/m, exchange stiffness A = 1.3 × 10^−11^ J/m and gyromagnetic ratio Y = 176 rad GHz/T. For calculations in ADLs we have reduced the theoretical thickness for 2 nm in reference to the nominal values. For this reduction, which can come from an oxidation process on the top face of ADL, we have got the best agreement between numerical and experimental results.

In order to select calculated modes corresponding to the most intensive modes visible in BLS measurements, we estimated the cross-section of BLS using the square modulus of main Fourier coefficient of the out-of-plane dynamical component of the magnetization in its expansion into the wave vector domain^54^.

## Additional Information

**How to cite this article**: Tacchi, S. *et al*. Universal dependence of the spin wave band structure on the geometrical characteristics of two-dimensional magnonic crystals. *Sci. Rep.* **5**, 10367; doi: 10.1038/srep10367 (2015).

## Figures and Tables

**Table 1:** Geometrical parameters of the ADL samples analyzed: periodicity- *a*, diameter of antidots - *D*, thickness - *t* and inter-hole separation (width of the effective nanowires) - *w*. The last column shows the ratio *t/w*.

**Series**	***D*** **(nm)**	***t*** **(nm)**	***w*** **(nm)**	***t/w***
S1 (*a* = 440 nm)	240	12	200	0.06
	240	20	200	0.10
	240	30	200	0.15
S2 (*a* = 470 nm)	355	12	115	0.10
	355	20	115	0.17
	355	30	115	0.26
S3 (*a* = 420 nm)	140	30	280	0.11
	180	30	240	0.13
	220	30	200	0.15
	260	30	160	0.19
